# Type I choledochal cyst. Total laparoscopic resection and Roux-en-Y reconstruction to two separated ducts

**DOI:** 10.1093/jscr/rjae543

**Published:** 2024-08-28

**Authors:** Natalia Reyes, Camila Sotomayor, Martín Inzunza, Eduardo Briceño, Eduardo Viñuela, Jorge Martínez, Nicolás Jarufe

**Affiliations:** Department of Hepatobiliary Surgery, Hospital Clínico Universidad Católica, Santiago 8330024, Chile; Department of Hepatobiliary Surgery, Hospital Clínico Universidad Católica, Santiago 8330024, Chile; Department of Hepatobiliary Surgery, Hospital Clínico Universidad Católica, Santiago 8330024, Chile; Department of Hepatobiliary Surgery, Hospital Clínico Universidad Católica, Santiago 8330024, Chile; Department of Hepatobiliary Surgery, Hospital Clínico Universidad Católica, Santiago 8330024, Chile; Department of Hepatobiliary Surgery, Hospital Clínico Universidad Católica, Santiago 8330024, Chile; Department of Hepatobiliary Surgery, Hospital Clínico Universidad Católica, Santiago 8330024, Chile

## Abstract

A choledochal cyst is a rare condition that requires surgical treatment to prevent complications, such as obstructive jaundice, cyst rupture, cholangitis, and the risk of malignancy. Complete cyst excision is considered the best option, as it reduces the risk of inflammation and the development of cholangiocarcinoma. Therefore, cholecystectomy and complete cyst resection followed by reconstruction with a Roux-en-Y hepaticojejunostomy is the treatment of choice. We present a case (with video) that shows the complete resection of a type I choledochal cyst with Roux-en-Y reconstruction of two separate ducts since the right posterior duct reached the cyst independently. The laparoscopic approach offers all the advantages of mini-invasive surgery and better visualization of the structures; however, biliary reconstruction to fine ducts implies a surgical challenge that requires high training in mini-invasive surgery.

## Introduction

Choledochal cyst (CC) is a rare congenital pathology that causes dilation of the extrahepatic, intrahepatic biliary tree, or both. Its incidence varies between 1 in every 13 000 and 2 000 000 live births [1], with type I CC representing 80%–90% of these cases [2]. Symptoms appear in 84.5% of patients, with abdominal pain and jaundice being the most prevalent. Complications include cholelithiasis, cholangitis, spontaneous rupture, pancreatitis, and cholangiocarcinoma [3].

Complete resection of type I CC involves removing the entire dilated extrahepatic biliary tree from the confluence to the intrapancreatic common bile duct to eliminate the risk of malignant transformation. Reconstruction is generally performed using Roux-en-Y hepaticojejunostomy (RYHJ). The association between an anatomical variant of the right posterior hepatic duct (RPHD) and CC is rare, with few documented cases in the medical literature [4].

We present the case of a patient with a type I CC resolved by laparoscopic surgery, with complete resection of the cyst and Roux-en-Y reconstruction of two separate ducts since the right posterior duct reached the cyst independently. Our goal is to contribute to the limited evidence regarding this rare case, as a few published cases in the medical literature have undergone this surgical technique treatment.

## Case report

The present case corresponds to a 45-year-old woman with a history of recurrent episodes of recurrent abdominal pain and jaundice, which has required sporadic hospitalizations, during which she underwent endoscopic retrograde cholangiopancreatography with a bile duct stent placed.

A magnetic resonance cholangiopancreatography revealed the presence of a large 5 cm CC, classified as type I according to the Todani classification, accompanied by significant lithiasis of up to 3 cm inside and cholelithiasis. An anatomical variant was identified in which the RPHD drained directly into the CC.

The surgical team performed a complete resection of the CC, accompanied by reconstruction using two independent enterotomies and anastomoses, given the considerable distance between the right anterior and posterior bile ducts. The common hepatic duct (CHD) was dissected distally until the intrapancreatic bile duct of normal caliber was reached. Proximally, dissection continued to the confluence of the right anterior and left ducts. Two separate hepaticojejunostomy were performed, first to the right posterior duct and then to the CHD (see Video 1).

The patient showed a favorable recovery with no signs of biliary fistula, allowing the drain to be removed, and the patient to be discharged 5 days postoperatively. Follow-up revealed no stenosis of the hepaticojejunostomy, and the patient reported no significant discomfort.

## Discussion

CC is a rare pathology in adults. Although mainly diagnosed during childhood, 20%–25% of patients are diagnosed in adulthood. It affects 0.1% of the population and is four times more common in women [5]. The incidence of malignant transformation to cholangiocarcinoma is 3%–5%, increasing from 0.7% in the first decade to over 14% after 20 years [1]. Therefore, early diagnosis and cyst resection surgery are essential.

The evolution of surgical treatment has progressed from cyst-enteral anastomosis, associated with symptom recurrence and malignancy risk, to complete cyst resection with biliodigestive reconstruction via open or laparoscopic approaches [6].

Currently, minimally invasive approaches are used to treat CC, as laparoscopy provides better visualization and more precise manipulation. Zhen *et al.* compared the open and laparoscopic techniques, finding that laparoscopy resulted in a shorter hospital stays (MD = −1.93; 95%CI = −2.51 to −1.36; *P* < .00001) and faster recovery of intestinal function (MD = −0.94; 95%CI = −1.33 to −0.55; *P* < .00001), with no significant differences in postoperative complications (RR = 1.04; 95%CI = 0.66–1.62; *P* = .88). Although the laparoscopic group had longer operative times (MD = 56.57; 95%CI = 32.20–80.93; *P* < .00001), it had lower rates of red blood cell transfusion (RR = 0.20; 95%CI = 0.11–0.38; *P* < .00001) and intestinal obstruction (RR = 0.17, 95%CI = 0.04–0.77; *P* = .02) [7].

The laparoscopic technique represents a surgical challenge that requires advanced experience and skills from the surgical team.

The standard biliodigestive reconstruction technique is RYHJ. However, studies have defended hepaticoduodenostomy as a more physiological tension-free technique, faster to perform, and with potential for future endoscopic interventions. Ai *et al.* identified that patients undergoing biliodigestive reconstruction with hepaticoduodenostomy had a shorter hospital stay (MD = −0.40; *P* = .02), a shorter operative time (MD = −59.54; *P* < .00001), and a lower incidence of adhesive intestinal obstruction (OR = 0.20; *P* = .02) than those undergoing hepaticojejunostomy. However, it was associated with a higher incidence of gastritis (OR = 6.24; *P* = .002) [8]. Howell *et al.* [9] reported that patients undergoing hepaticojejunostomy had a lower readmission rate compared to those undergoing hepaticoduodenostomy (4.0% vs. 10.5%, OR = 0.34, CI [0.12, 0.79], *P* = .02), the causes of readmission being pancreatitis, surgical site infection, abdominal pain, and cholangitis.

Regarding the reconstruction technique of the anatomical variant of the RPHD, there are two approaches depending on the distance between the CHD and the RPHD: ductoplasty and double hepaticojejunostomy [4]. Ligation or failure to identify the RPHD during surgery can lead to complications such as cholangitis, liver abscess, liver atrophy, cirrhosis, biliary fistula, or biliary peritonitis [10].

There is little evidence comparing both techniques, the majority being in the pediatric population. Diao *et al.* [11] evaluated laparoscopic treatment for aberrant hepatic duct (AHD) in children with CC, identifying that the AHD was frequently confused with adhesions, causing bile leaks in the postoperative period. Xie *et al.* [12], when comparing robotic versus laparoscopic surgery for CC in children with AHDs, found that of a total of 22 patients, the majority required ductoplasty and only two required double hepaticojejunostomy in both groups, with no significant differences in postoperative complications. Bile leak is one of the most worrying complications associated with this procedure [4].

In this case, the technique of choice was separate hepaticojejunostomy, given the distance between both ducts, as the evidence suggests. It is essential to mention that to perform this procedure, a specialized team with the experience and skills necessary to perform fine anastomoses laparoscopically was required, and adequate training was necessary to achieve good results.

It is important to note that little additional published evidence resembles this case. We hope to contribute to medical knowledge to manage these challenging cases effectively.

## Supplementary Material

CCtypeI_rjae543
